# Treatment of Scarlet Fever and Scarlatinous Dropsy by Baths

**Published:** 1871-05

**Authors:** 


TREATMENT OF SCARLET FEVER AND SCARLATINOUS DROPSY BY BATHS.—In twelve cases of scarlet fever, complicated with diphtheria, the cold-water bath was employed whenever the temperature of the child reached from 38·5° C. to 39·5° C. The temperature of the bath was 25° C., and the time of immersion varied from eight to ten minutes, the bath being repeated, in some cases, every hour. Of these cases five died, while seven recovered; while in no case did dropsy supervene. Cases of scarlatinous dropsy were treated according to Liebermeister's suggestion, by warm baths, the temperature of which was gradually raised from 38° to 40° C. The child was kept in the bath half-an-hour, and copious diaphoresis kept up for two hours. The result was very satisfactory, the transudation being gradually and progressively absorbed without other medication. —*Jahrb fur Kindersheilkunde.*

